# Achalasia Combined with Idiopathic Gastroparesis Treated with Peroral Endoscopic Myotomy and Gastric Peroral Endoscopic Myotomy in a Single Procedure

**DOI:** 10.5152/tjg.2023.23276

**Published:** 2023-09-01

**Authors:** Yating Wang, Chunxi Zhang, Dongqiang Zhao

**Affiliations:** Department of Gastroenterology, The Second Hospital of Hebei Medical University, Shijiazhuang, Hebei, China

## Case Presentation

Achalasia combined with idiopathic gastroparesis is an exceedingly uncommon medical condition, and its underlying mechanisms remain enigmatic. We report a rare case of a 19-year-old female patient who suffered from dysphagia accompanied by regurgitation for 3 years, with worsening symptoms over the last 2 months. She also complained of postprandial fullness and mild bloating. Previous treatment with medication failed to provide effective relief. High-resolution manometry (HRM) confirmed type I achalasia (Figure 1). The gastric emptying study (GES) confirmed the delayed gastric emptying. Barium esophagram revealed esophageal dilation and the passage of contrast into the gastric chamber through a filiform pathway. Additionally, spasm-like contractions of pylorus were observed (Figure 2). Endoscopy revealed the failure of lower esophageal sphincter to relax properly and spastic pylorus. After ruling out potential causes, it is considered that the gastroparesis is idiopathic. Given the complexity of the case and the refractory symptoms, peroral endoscopic myotomy (E-POEM) and gastric peroral endoscopic myotomy (G-POEM) in one procedure was chosen as the treatment modality. This approach would address the achalasia and idiopathic gastroparesis simultaneously. To date, there have been few reported cases about dual-POEM (E-POEM + G-POEM).^1- 3^

## Technique

Peroral endoscopic myotomy was conducted first to facilitate cardia dilation, thus minimizing the mucosal injury to the cardia caused by the endoscopic shaft during G-POEM. The procedure was carried out using therapeutic endoscope equipped with a water-jet function (GIF-Q260J; Olympus, Tokyo, Japan). The patient was given general anesthesia with endotracheal intubation. First, a submucosal space was created using 10% glycerin solution supplemented with 0.3% indigo carmine at 10 cm above the gastroesophageal junction. A 2 cm longitudinal mucosal incision was then made using the DualKnife (KD-655L 2.0 mm; Olympus). The endoscope was then introduced into the submucosal space. A submucosal tunnel was then created and carefully extended 2 cm distal to the cardia. Asymptotic full-thickness myotomy was performed with DualKnife to a point 2 cm below the cardia, 7 cm in length. The mucosal closure was carried out with clips. After E-POEM, submucosal injection was performed in the greater curvature; 7 cm upstream of the pylorus, a 1.5 cm mucosal incision was then made. A submucosal tunnel was created along the greater curvature toward the pylorus. Stepwise and deep pyloromyotomy was performed. Finally, the mucosal incision was closed with clips. Intravenous antibiotic cefamandole were administered after dual-POEM (Video 1).

On the fifth postoperative day, the barium esophagram indicated a rapid passage of contrast from esophagus into the stomach and then to the duodenum (Figure 3). The patient was then discharged on an oral diet after 2 weeks of hospital stay, and her symptoms had improved greatly (Eckardt score decreased from 7 to 0; Gastroparesis Cardinal Symptom Index reduced from 2 to 0.22). Follow-ups were carried out 6 months and 1 year post operation, and the patient reported no dysphagia or regurgitation.

## Conclusion

It is worth noting that patients diagnosed with esophageal dysmotility are more likely to exhibit gastric dysmotility and vice versa. The exact relationship between these conditions remains unknown, but shared underlying pathophysiological mechanisms, such as autoimmune factors, genetic predisposition, or neurogenerative processes, may play a role. Further research is needed to elucidate the underlying mechanisms and clinical implications.

^*^The video file linked to this article is available in the online version of the journal. Or you can utilize the QR code provided on this page to gain access.


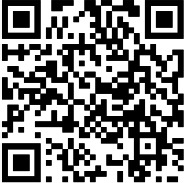


## Figures and Tables

**Figure 1.**
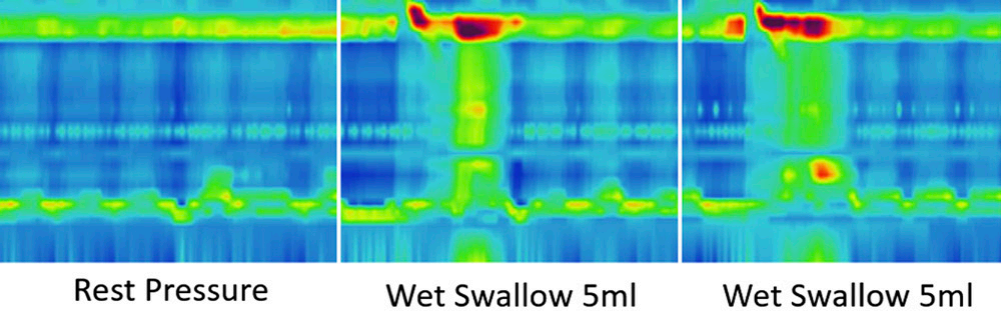
High-resolution manometry: type I achalasia.

**Figure 2.**
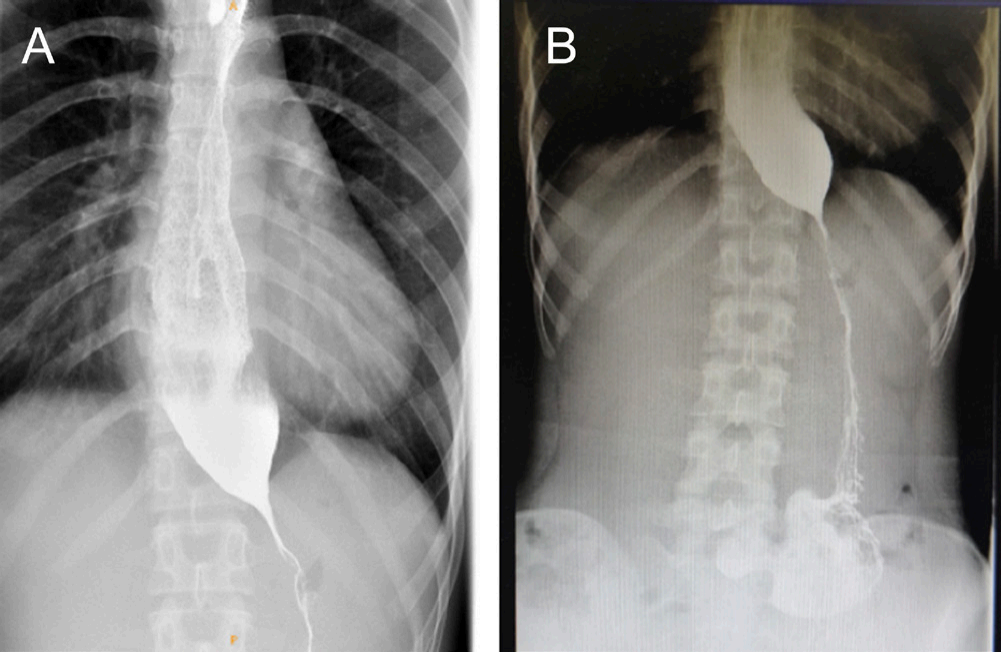
Preoperative esophagography. (A) Dilation of the esophagus and filiform passage of the contrast into the gastric chamber. (B) Spasm-like contractions of pylorus.

**Figure 3.**
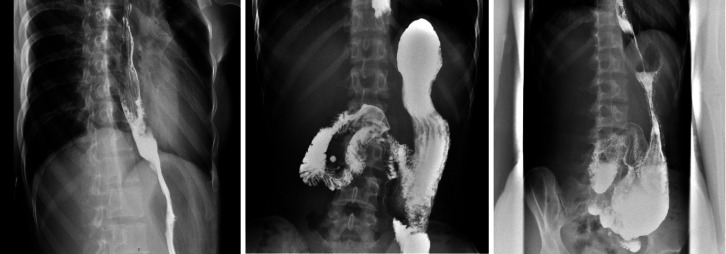
Postoperative esophagography indicated a rapid passage of contrast from esophagus into the stomach and then into the duodenum.
